# Double-plating of ovine critical sized defects of the tibia: a low morbidity model enabling continuous in vivo monitoring of bone healing

**DOI:** 10.1186/1471-2474-12-214

**Published:** 2011-09-29

**Authors:** Joachim A Hahn, Tanja S Witte, Daniel Arens, Alexandra Pearce, Simon Pearce

**Affiliations:** 1 AO Research Institute, Clavadelerstrasse 8, Davos, Switzerland

## Abstract

**Background:**

Recent studies using sheep critical sized defect models to test tissue engineered products report high morbidity and complications rates. This study evaluates a large bone defect model in the sheep tibia, stabilized with two, a novel Carbon fibre Poly-ether-ether-ketone (CF-PEEK) and a locking compression plate (LCP) which could sustain duration for up to 6 month with an acceptable low complication rate.

**Methods:**

A large bone defect of 3 cm was performed in the mid diaphysis of the right tibia in 33 sheep. The defect was stabilised with the CF - PEEK plate and an LCP. All sheep were supported with slings for 8 weeks after surgery. The study was carried out for 3 months in 6 and for 6 months in 27 animals.

**Results:**

The surgical procedure could easily be performed in all sheep and continuous in vivo radiographic evaluation of the defect was possible. This long bone critical sized defect model shows with 6.1% a low rate of complications compared with numbers mentioned in the literature.

**Conclusions:**

This experimental animal model could serve as a standard model in comparative research. A well defined standard model would reduce the number of experimental animals needed in future studies and would therefore add to ethical considerations.

## Background

The healing of large bone defects caused by tumour, infection or high energy trauma remains one of the most challenging orthopaedic problems for both surgeons and patients. Autologous bone transplantation using cancellous bone graft harvested from sites such as the iliac crest, is currently considered as "gold standard" for reconstruction of these defects [1,2]. The process of harvesting the cancellous bone, however, is associated with a high rate of donor site morbidity, and an increased risk of infection, nerve damage and loss of function [3]. Furthermore, the amount of suitable bone can be limited, particularly in older patients. Tissue engineering offers a possible solution to the problems of autologous bone transplantation. Tissue engineered constructs employ a combination of biodegradable, osteoconductive scaffolds, cells that provide osteogenic potential and osteoinductive growth factors [4,5]. Comprehensive evaluation of new tissue engineered constructs requires both in vitro and in vivo testing. In vitro testing is popular for the characterisation of tissue engineered constructs particularly as medical researchers embrace the principles of animal reduction. In vitro testing gives useful information regarding cytotoxicity, genotoxicity, cell proliferation and differentiation [6,7] and is particularly useful for the screening of new materials [8]. The final stages of evaluation prior to use in humans, however, requires in vivo testing in an animal model in order to demonstrate the tissue response to the construct. Critical sized defect models that simulate the poor healing seen in human patients with large bone defects have been developed in a number of species including rodents, rabbits, pigs, goats, dogs and sheep [9,10]. The sheep critical sized defect model offers some unique advantages over other species that makes it particularly feasible for modelling human orthopaedic disease. Adult sheep are similar in body weight to humans and have long bones with similar surface to volume ratios and remodelling properties to humans. Therefore, the biomechanical and bone healing characteristics of sheep are more similar to those found in humans than of other, smaller species [9]. Furthermore, sheep are easy to handle, inexpensive to keep, and are not considered companion animals, which improves their ethical acceptability. A number of studies using sheep critical sized defect models report high morbidity and complication rates that range from 2.5% [11] up to 33% [12]. These studies vary in their experimental design, surgical technique and defect stabilisation technique, which makes comparison between these studies difficult. For this reason and after experiencing high complication rates in previous experiments in our institute we have designed a critical sized defect model in the sheep using two plates that enables in vivo radiographic monitoring of bone healing while overcoming the problems of high morbidity and high complication rates. Therefore, we set out to test the hypothesis that a 3 cm, critical sized defect in the sheep tibia stabilized with two, a novel Carbon fibre Poly-ether-ether-ketone (CF-PEEK) and a locking compression plate (LCP), can sustain a duration of up to 6 month and allows in vivo monitoring of bone healing.

## Methods

### Experimental animals

Thirty-three, skeletally mature, Swiss alpine sheep were selected for the study. The mean weight of the sheep was 62.6 kg (range: 50.0 to 78.0 kg). A pre-operative physical examination revealed no gross abnormalities in any of the sheep. Pre-operative radiographs which were used to confirm skeletal maturity based on closure of the tibial physis, revealed no bony abnormalities of both tibiae. Six sheep were maintained post-operatively for duration of 3 months and 27 sheep were maintained for duration of 6 months. Following surgery, sheep were housed in single stalls, fed a maintenance ration of hay and had *ad libitum *access to water for the whole duration of the study. All procedures during the study were carried out according to the Swiss Laws of animal welfare and were approved by the local Ethics Commission of the official veterinary authorities (Authorisation Number: 10/2005).

### Surgical Procedure

All surgical procedures were performed under general anaesthesia. General anaesthesia was induced with butorphanol (0.06 mg/kg BW intravenously (iv), Morphasol, Gräub AG, Bern, Switzerland), midazolam (0.3 mg/kg BW iv, Dormicum, Roche Pharma, Reinach, Switzerland) and thiopental (5-10 mg/kg BW iv to effect, Pentothal, Abbott AG, Cham, Switzerland). Sheep were intubated and anaesthesia was maintained with 2% isoflurane (Pharmacia and Upjohn AG, Dübendorf, Switzerland) in oxygen.

**Figure 1:**
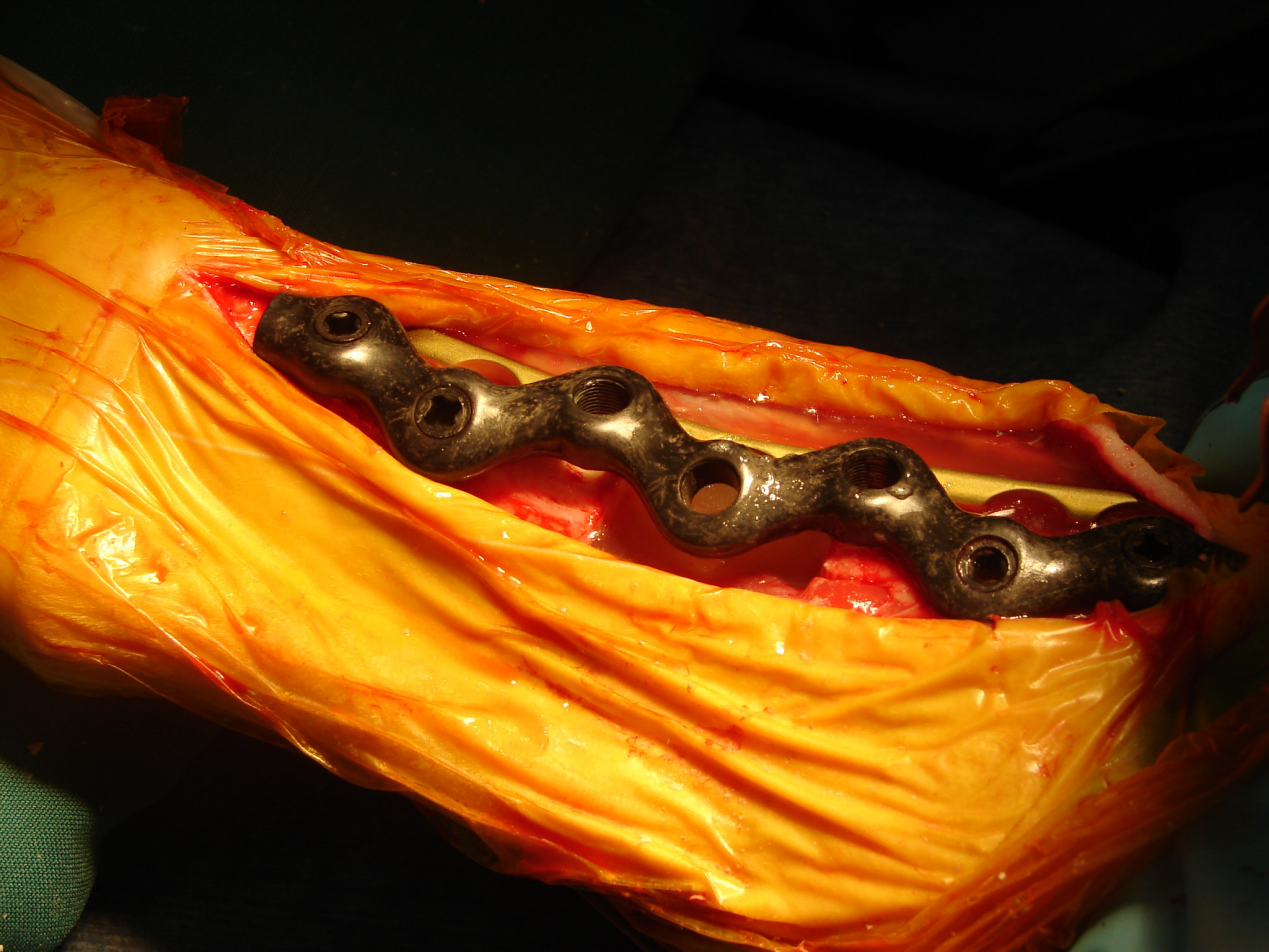
**Intra-operative situation after ostectomy and osteosynthesis**. The image shows the cranial LCP and the medial snakeplate on the tibia. The ostectomy in the tibia is clearly visible leaving space for potential tissue engineered solutions.

**Figure 2:**
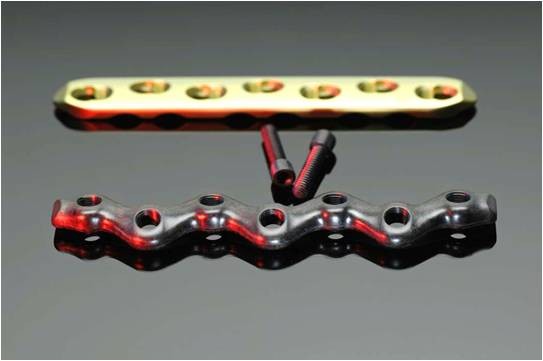
**The two osteosynthetic plates used**. Upper: 7 hole broad LCP; lower: CF - PEEK (snakeplate) with the corresponding screws.

**Figure 3:**
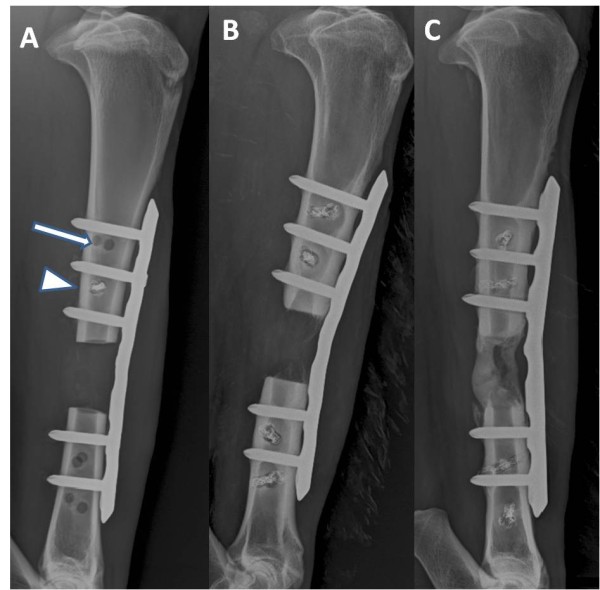
**Continuous radiographic evaluation in vivo**. The latero-medial radiographic image of the right tibia shows the situation two weeks post operative (Figure 3A). The cranial LCP is clearly visible while the medial snakeplate is radiolucent. The screws of the snakeplate randomly include tantalum wires (note the white arrow head) which make them visible on radiographs. If no tantalum wires are included in the implant the screw is completely radiolucent (image A, white arrow). On the radiographic image 24 weeks post operative (Figure 3B) very little bone formation is visible. The cortices alongside the ostectomy gap have narrowed and no bridging has occurred. Even though in this case no load sharing occurred there are no signs of implant loosening or instability. On a radiographic image 24 weeks post operative evaluating a different tissue engineered solution (Figure 3C) bridging has occurred. It was possible to evaluate the amount of bone formation during the study in one plane.

Each sheep was placed in right lateral recumbence and the right tibia was prepared for aseptic surgery by routine clipping and cleaning of the skin using a chlorhexidine solution (Hibiscrub, Swissmedic, Bern, Switzerland). The right tibia was draped routinely for a medial approach to the tibial diaphysis. A 15 cm longitudinal incision centred on the mid diaphysis was made through the skin and subcutaneous tissues. Care was taken to avoid cutting the periosteum. At the level of the mid diaphyses (7 cm above the medial malleolus of the tibia) a length of 3 cm was marked on the bone. A seven hole, locking CF - PEEK plate (snakeplate, Icotec AG, Altstätten, Switzerland; Figure 1 and 2) was placed. Using the corresponding drill sleeves, the first, second, sixth and seventh holes were pre-drilled with a 4.0 mm drill bit. The third, fourth and fifth holes were left empty as they were within the defect zone. The plate was then removed and a 2.7 cm custom made spacer was placed and fixed with a monocortical 3.5 mm screw in the centre of the marked, pre-determined defect zone. Using an oscillating saw, the full diameter of the tibia at the distal end of the spacer was transected under constant cooling with Ringer's solution (B. Braun Melsungen AG, Melsungen, Germany). The tibia was then transected at the proximal end of the spacer and the 3 cm defect created in removing of the bone segment with the spacer attached. Care was taken to ensure that all of the periosteum was removed and that no tags of periosteum remained at the transected bone ends. The snakeplate was then placed on the medial aspect of the tibia again, the pre-drilled screw holes were tapped and 4.5 mm CF - PEEK screws were placed. A random selection of the screws contained tantalum fibres that enabled them to be seen radiographically (Figure 3A). The screws were tightened with a maximum force of 1.5 Nm. Subsequently, a 7-hole, 4.5 mm locking compression plate (LCP, Synthes GmbH, Oberdorf, Switzerland) was contoured to the cranial aspect of the tibia. The first, second, third, sixth and seventh holes were drilled using a 4.3 mm drill bit and self-tapping 5 mm screws (Synthes GmbH, Oberdorf, Switzerland) were placed. Care was taken to ensure that the plates were placed 90° to each other, that all screws were placed bicortically, and that the screws did not interfere with each other. This was done under visual control and resulted in a 3 cm mid diaphyseal defect with the periosteum completely removed. The appropriate treatment was placed into the defect and the fascia and subcutis were closed with continuous sutures (Polyglecaprone 25, Ethicon Inc., Neuchatel, Switzerland). The skin was closed using skin staples.

### Postoperative management and monitoring

Postoperative analgesia was provided by intramuscular (im) administration of carprofen (4 mg/kg BW, Rimadyl, Pfizer AG, Zürich, Switzerland) and subcutaneous (sc) injections of buprenorphine (0.01 mg/kg BW, Temgesic, Reckitt and Colman Pharm., Hull, UK) at the end of surgery. Additional analgesia was administered by means of buprenorphine (0.01 mg/kg BW sc three times daily, Temgesic, Reckitt and Colman Pharm., Hull, UK) for 3 days and carprofen (4 mg/kg BW im once daily, Rimadyl, Pfizer AG, Zürich, Switzerland) for 5 days. No antimicrobial therapy was administered during or after aseptic surgical procedures.

**Figure 4:**
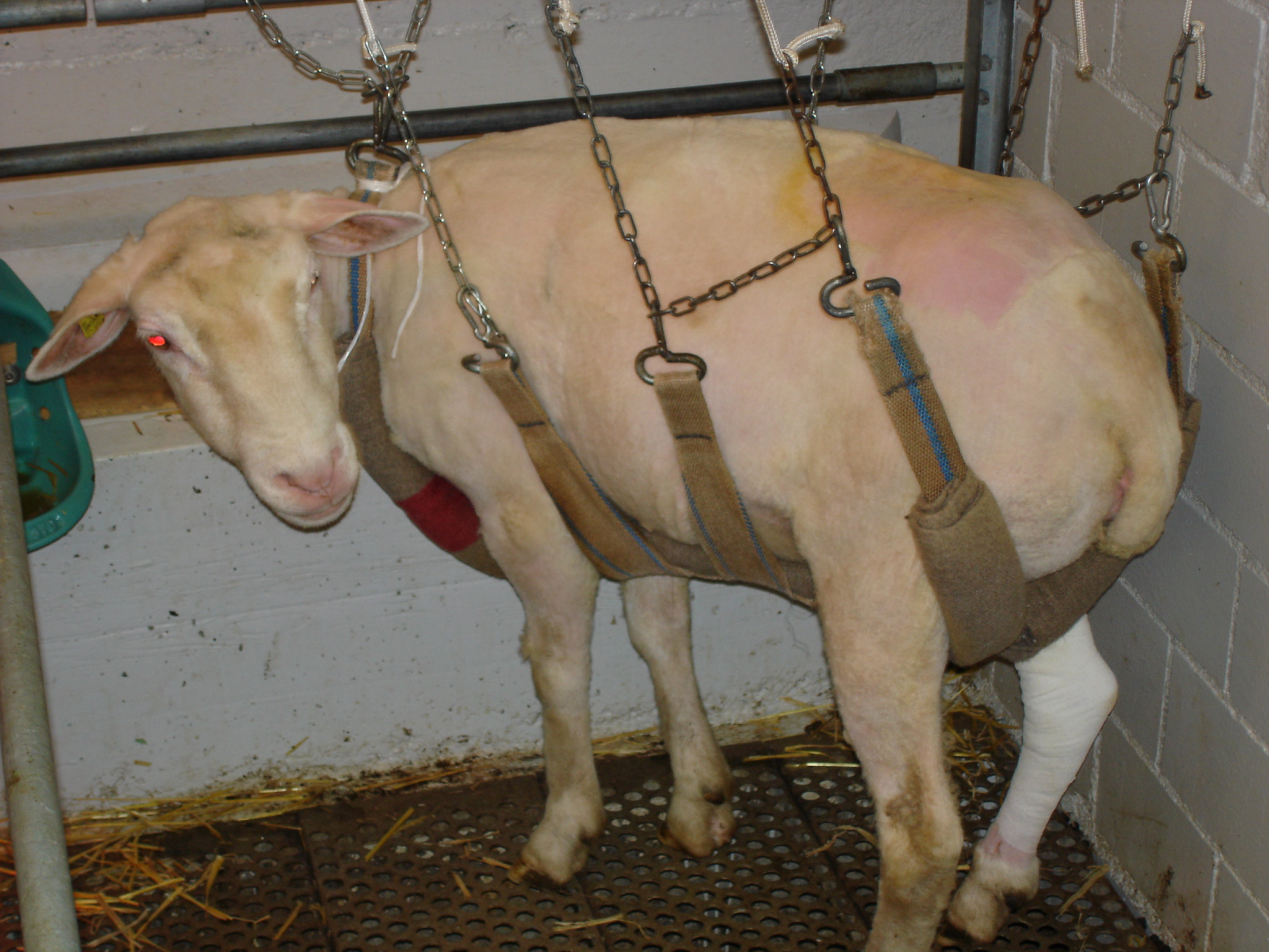
**Animal supported by slings**. A sheep on the second day after surgery. The slings allow the sheep to fully weight bear on the operated leg, but prevents peak loads during laying down and standing up.

After surgery sheep were housed in single stalls. For eight weeks post surgery the sheep were held in slings (Figure 4) allowing them to stand, bear full weight on all legs and move around in their boxes to a certain degree. During this time the sheep were protected them from high loading forces experienced when rising from a lying down position. This handling concept was pre-planned and defined in the animal experimental permission approved by the Ethics Comission. The sheep were monitored each day by an experienced animal caretaker and a veterinarian for signs of inflammation, lameness and general wellbeing. Here weight bearing and movement on the operated limb were controlled visually. Two weeks after surgery the skin staples were removed.

Lateromedial radiographs of the operated tibia were taken immediately after surgery and every second week (Figure 3). The radiographic examinations were performed under sedation using detomidine (0,03 mg/kg BW im, Domosedan, Pfizer AG, Zürich, Switzerland). Bone regeneration was quantified using a digital image software analysis programme (GIMP, GNU General Public Licence). The results of the radiographic evaluation are published elsewhere [13]. At the end of the study the sheep were euthanized by means of an iv barbiturate overdose (60 mg/kg BW, Pentobarbital, Eutha 77, Swissmedic, Bern, Switzerland). Time points of euthanasia were at 3 month or 6 month after surgery, respectively, depending on the original study question.

## Results

There were no intraoperative complications during the study. Surgical time was between 90 and 120 minutes. Anaesthesia and recovery phase were uneventful for all operated sheep. Twenty-four hours after surgery all sheep were fully weight bearing on the operated limb. At no time after surgery clinical signs of infection were noticed. At about 2 weeks after surgery no clinical signs of inflammation were detected with the incision wound healed. The sheep adapted well to the slings and no complications were associated with the use of the slings. The animals were taken out of the slings at 8 weeks regardless of the radiological state of healing within the defect.

**Figure 5:**
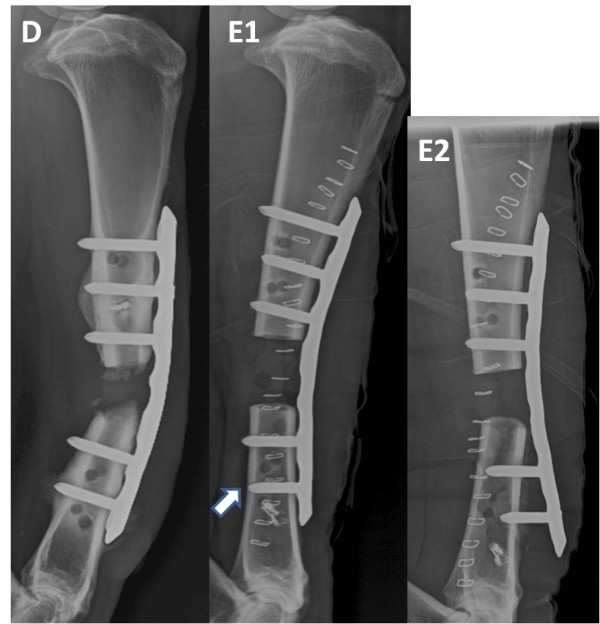
**Radiographic evaluation of complications that have occurred**. Radiographic image of a sheep that had to be euthanized 3 month after surgery (Figure 5D). Here both distal screws of the snakeplate were broken and the LCP started to bend. Radiographic image at the end of surgery (Figure 5E1), note screw number 7 in the LCP is not placed bicortical (white arrow). Radiographic image of the same sheep the day after surgery (Figure 5E2). At necropsy a spiral fracture extending through the 2 distal screw holes of the LCP was diagnosed.

Severe postoperative complications were experienced in 2 sheep. One sheep was euthanized one day after surgery due to a fractured leg (Figure 5). The immediate postoperative radiograph showed that the most distal screw in the LCP was too short to engage both cortices (Figure 5). At necropsy the distal screw had pulled out and a spiral fracture extending through the two distal screw holes of the LCP was diagnosed. A second sheep was euthanized after 3 months because screw numbers 6 and 7 of the snakeplate were broken which resulted in increased loading and subsequent bending of the LCP (Figure 5). The complication rate was comparable in both groups with 1 sheep in the 3 month and the 6 month group, respectively. Low grades of lameness were noted in two sheep at 3 and 4 months after surgery. In these sheep the lameness was localised to the front limbs and laminitis was suspected. Based on this diagnosis, the sheep were placed on thick straw bedding and the lameness resolved. No lameness or pain was detected in any of the other sheep during the whole study period. The overall incidence of severe complications was 2 out of 33 sheep (6.1%).

## Discussion

The successful treatment of large bone defects remains extremely challenging. New treatment modalities such as tissue engineered solutions are continuously being developed in order to expedite healing. Therefore, there is a need for reliable critical sized bone defect models in animals that can test the efficacy of these solutions. Because of the challenging nature of critical sized defects, many animal models described in the literature report high morbidity and high complication rates. For this reason we have developed a model that reduces morbidity and complications while enabling in vivo monitoring of bone healing.

Animal models described in the literature vary considerably, with differences in the species used, the defect size, ostectomy location, surgical technique and the type of osteosynthesis used. The defect size is generally determined by the dimensions of the bone in which the defect is made. In the sheep, a metatarsal defect of 0.6 cm constitutes a critical sized defect [14] whereas in the tibia defects of 3-4 cm are most common [10,11,15]. Unlike the femur which is limited by its thin cortices and its accessibility due to large amounts of overlying musculature, and the metatarsus which is limited in its vascularity due to scant overlying musculature [14], the tibia is almost two-thirds covered by muscle which provides excellent blood supply while at the same time having an easily accessible medial aspect which is devoid of muscle and major blood vessels. Furthermore, there is approximately 9.5 cm of bone spanning the mid tibial diaphysis that is almost circular in cross-section [3], making it particularly suitable for the placement of scaffolds. Thus, the dimensions of the tibia make it feasible to use either human or veterinary implants for stabilisation of the defect.

Using the double plating technique, 6.1% of the sheep in this study experienced severe complications necessitating euthanasia. One sheep was euthanized due to a fractured tibia the day after surgery. Post mortem evaluation revealed that the surgical technique may have contributed to failure in this case. The results, in which we had one complication between 0 months and 3 months and one complication between 3 months and 6 months, are comparable with the low complication rate reported by Den Boer et al [11] following the use of an interlocking nail for fixation of a 3 cm tibial defect for a period of 3 months. Whereas, the overall complication rate of 6.1% in the present study is more favourable than those experienced in our own institute using a different sheep tibial defect model [12]. It is difficult, however, to directly compare complication rates of different studies because these often show variability in defect size, study duration and type of fixation. Interestingly, many of the studies which use the sheep tibial defect have duration of less than 6 months. In an earlier study in our own institute we performed a single medial LCP osteosynthesis with a 4 cm mid tibial defect in the sheep. In this study, five of 15 (33%) animals of the experimental group did not reach the termination of the study at 3, 6 and 10 month due to implant failure [12]. This highlights the value of the double plating technique in providing maximum stability over a prolonged period of time.

Other types of double fixation used for sheep tibial defects have demonstrated higher complication rates than the present study. Gao et al reported an 8.3% complication rate following the use of a two-plate fixation technique for a 1.6 cm tibial defect over a period of 4 months [16]. Krischak et al found that 2 out of 9 sheep in which a two-plane external fixation was used experienced distal fractures through the pin tracks [17]. This is a complication rate of 22%. Also, 4 out of the 9 sheep experienced deep infections; a complication that is commonly associated with the long-term use of external fixation devices. In the study by Krischack et al, healing of the osteotomy was reduced when compared to a one-plane fixation [17]. The authors suggest that this might be due to greater disturbance of the blood supply when using a two-plane fixation [17]. Interference between the fixation device and the blood supply and also with the implanted scaffold must be taken into consideration when designing a sheep critical sized defect study. The use of conventional dynamic compression plates (DCPs) can result in impaired blood supply to the bone due to compression of the periosteum. In contrast, angular stable implants such as the plates used in the present study, do not require direct contact to the bone and do not compress the periosteum. Consequently, the major blood supply to the bone is preserved. Intramedullary nailing is another form of fixation [11,15] that results in complication rates that tend to be quite low; however, there are several disadvantages to this fixation method. Damage to the intramedullary blood supply as a result of reaming may delay healing. To make matters more complicated, the nail itself may affect bone healing, such as in the case of titanium nails which are known to have good osteoconductivity. The presence of the nail, which occupies space within the centre of the defect, limits the amount of test material that can be implanted and also affects the shape of the scaffold material in order to accommodate the centrally positioned nail. Furthermore, the presence of the nail can interfere with mechanical testing of the explanted bones and continuous radiographic evaluation of bone formation is difficult. Two plate constructs [16] often limit the ability for continuous in vivo evaluation.

In the current study using a two-plate system, in which one plate was radiolucent, continuous evaluation of bone and callus formation was possible by taking latero-medial radiographic images (Figure 3). This enables the researcher to evaluate the amount of callus formation in different experimental groups as well as the onset of callus formation. It has to be mentioned, however, that the snakeplate can not be contoured and the maximal loading strength is inferior to titanium alloy implants. This may have contributed to the failure of the distal screws in the second sheep that had to be euthanized after 3 month.

The time frame up to 3 month was conducted to test the biocompatibility of the tissue engineered solutions since the occurrence of foreign body or inflammatory reactions to the material is indicative for this phase. In the time frame of 6 months, in previous studies dealing with similar critical sized defects of the tibia we have not found new bone formation after this time period. Most of the tissue engineered constructs tested except calcium phosphates were resorbed and replaced by new bone within this period. The rigid internal fixation using two angular stable plates could well lead to a late onset of healing as well as incomplete ossification due to load sharing situations between implants and bone. However, we believe that minimizing micro motions is ideal for an animal model because these micro motions account for bone healing in an unknown amount. The repeatability of the present fixation method enables a reproducible rigidity in this model.

In hind side, the use of the slings in this critical sized defect model is questionable. After experiencing high complication rates in critical sized defect models in large animals in our own institute we have decided to accept the additional reduction of life quality for the sheep in order to lower the mortality significantly. All sheep were taken out of the slings after 8 weeks regardless the stage of healing. Even sheep that showed no or low degrees of bone healing without additional stability did not show any complications later on. However, it is possible, that micro fractures occurring during surgery have healed by this time point. Whether the use of the slings added significantly to the low complication rate in this study is not applicable at this time point and needs further investigation.

In total, 33 sheep were operated using this technique with a low complication rate. The use of the radiolucent snakeplate enabled continuous radiographic monitoring which was beneficial for evaluating the healing response over time.

## Conclusions

We have found the described model to be a simple and reproducible model for evaluating bone healing continuously throughout the duration of the study. It results in low morbidity and a low complication rate. Therefore, it is sustainable for at least 6 months even when no load sharing between implant and bone occurs. For these reasons we believe that this model can be used as a standard model for the evaluation of new tissue engineered solutions for the treatment of large bone defects.

## Competing interests

The authors declare that they have no competing interests.

## Authors' contributions

JH participated in the design of the study, carried the surgeries, participated in the evaluation of the bone healing and wrote the manuscript. TSW was involved in the surgeries, wrote and critically revised the manuscript. DA participated in the design of the study and was involved in finalizing the manuscript. AP participated in drafting and revising the manuscript. SP participated in the design of the study, carried out a part of the surgeries, supervised the project and revised the manuscript. All authors read and approved the final manuscript.

## Pre-publication history

The pre-publication history for this paper can be accessed here:

http://www.biomedcentral.com/1471-2474/12/214/prepub
